# Improving sperm selection strategies for assisted reproduction through closing the knowledge gap in sperm maturation mechanics

**DOI:** 10.1093/hropen/hoaf040

**Published:** 2025-07-03

**Authors:** Hanah May Hart, Brett Nixon, Jacinta Hope Martin, Robert John Aitken, Geoffry Nunzio De Iuliis

**Affiliations:** Centre for Reproductive Science, School of Environmental and Life Sciences, College of Engineering, Science and Environment, University of Newcastle, Callaghan, NSW, Australia; Infertility and Reproduction Research Program, Hunter Medical Research Institute, New Lambton Heights, NSW, Australia; Centre for Reproductive Science, School of Environmental and Life Sciences, College of Engineering, Science and Environment, University of Newcastle, Callaghan, NSW, Australia; Infertility and Reproduction Research Program, Hunter Medical Research Institute, New Lambton Heights, NSW, Australia; Centre for Reproductive Science, School of Environmental and Life Sciences, College of Engineering, Science and Environment, University of Newcastle, Callaghan, NSW, Australia; Infertility and Reproduction Research Program, Hunter Medical Research Institute, New Lambton Heights, NSW, Australia; Centre for Reproductive Science, School of Environmental and Life Sciences, College of Engineering, Science and Environment, University of Newcastle, Callaghan, NSW, Australia; Infertility and Reproduction Research Program, Hunter Medical Research Institute, New Lambton Heights, NSW, Australia; Centre for Reproductive Science, School of Environmental and Life Sciences, College of Engineering, Science and Environment, University of Newcastle, Callaghan, NSW, Australia; Infertility and Reproduction Research Program, Hunter Medical Research Institute, New Lambton Heights, NSW, Australia

**Keywords:** assisted reproductive technologies, capacitation, male infertility, spermatozoa/sperm, sperm DNA quality, sperm function, sperm selection, sperm–zona pellucida binding, zona pellucida

## Abstract

**BACKGROUND:**

Male factors contribute to ∼50% of all infertility cases globally and are a major contributor to escalating use of ART. In most instances, sub-fertile men retain the ability to produce spermatozoa, albeit with reduced quality and function. By necessity, an important feature of ART is the use of technologies that bypass the natural selection barriers that prevent poor-quality spermatozoa from participating in fertilization. This means that ART carries a significant risk of facilitating fertilization with poor-quality gametes harbouring undetected DNA damage and/or altered epigenomes. Such a scenario may account for the epidemiological links between the use of ‘high intervention’ technologies [e.g. ICSI] and an increased risk of adverse offspring outcomes. Such data highlight a pressing need for improved sperm selection tools that better mimic natural selection barriers, to ensure only the highest-quality spermatozoa are used for ART.

**OBJECTIVE AND RATIONALE:**

Current sperm selection techniques for ART and the processes underpinning sperm maturation have often been considered independently and therefore reviewed separately. Here we outline the requirement for connecting research paradigms towards advancing clinical outcomes. This review highlights the importance of combining our advancing knowledge of sperm maturation biology with the pursuit of rational sperm selection strategies for the clinic; specifically, this narrative review summarizes the current clinical technologies used for sperm selection with a focus on their physiological relevance and limitations. We have given consideration to the events associated with sperm maturation and the importance of zona pellucida (ZP) binding as inspiration to inform the development of the next generation of sperm selection strategies. The connections and information presented should provide utility and interest for both clinicians and reproductive biologists alike.

**SEARCH METHODS:**

The PubMed database was queried using the keywords: sperm selection/function/DNA quality/epigenome, ART, ICSI, male infertility, capacitation, zona pellucida, sperm–zona pellucida binding, DNA damage, and biofabrication. These keywords were combined with other relevant phrases. Literature was restricted to peer-reviewed articles in English (published between 1972 and 2024) with references within these articles also searched.

**OUTCOMES:**

During natural conception, high-quality sperm are ‘selected’, maximizing the chances of fertilization with healthy gametes carrying intact genomic/epigenetic cargo. This sub-population of spermatozoa possess the capacity to interact with the female reproductive tract and complete the suite of functional maturation processes required for successful fertilization and initiation of embryonic development. However, ART ‘high intervention’ strategies bypass these selective barriers leading to an increased risk of inadvertently transferring genomic defects to the offspring with potential downstream consequences for offspring health. This review contextualizes why current sperm selection technologies have provided only minor improvement to live birth rates following ART. We posit that capitalizing on sperm–ZP binding (the penultimate step of successful fertilization) with novel ZP mimetic technologies provides an attractive, but understudied, tool for clinical selection of fertilization-competent spermatozoa for ART improvement.

**LIMITATIONS, REASONS FOR CAUTION:**

The risk of bias in the interpretation of findings for a narrative review cannot be completely eliminated. Literature was limited to the language the authors speak: English.

**WIDER IMPLICATIONS:**

ART has provided transformative advancement for infertile couples, however, gaps in our fundamental understanding of how the best gametes are ‘selected’ during natural conception, which when unaccounted for during clinical conception, present a risk of continued reliance on ART and health consequences for the next generation. The purpose of this article was to contextualize our current knowledge across both sperm maturation events and current selection strategies for these cells in the clinic. We outline the potential for improved clinical outcomes through the advancement of our understanding in gamete biology in concert with the development of novel methods for artificial gamete selection.

**STUDY FUNDING/COMPETING INTEREST(S):**

No external funding, but financial support was received from the School of Environmental and Life Sciences, University of Newcastle, Australia. R.J.A. is a scientific advisor to Memphasys Ltd, a biotechnology company with interests in reproductive health and responsible for developing the Felix™ electrophoretic sperm isolation device. R.J.A. receives salary and grant from, and has stock in, Memphasys Ltd. The other authors declare no conflicts of interest.

WHAT DOES THIS MEAN FOR PATIENTS? ART encompasses a suite of protocols designed to aid conception for those unable to achieve a natural pregnancy. Since the birth of the first baby conceived using ART in 1978, the uptake of these technologies has increased at an exponential rate. Despite its utility, a common misconception is that ART offers a simple, risk-free cure for all forms of infertility. Regrettably, this is not the case. Such risks, in combination with the significant number of ART cycles performed annually, highlights a pressing need for continued research into the causes and treatments for infertility, towards both improving conception rates and, mitigating associated reproductive and health risks. Here, we consider the suite of technologies currently available to ‘treat’ male infertility and offer guidance on future improvement strategies based on the sperm maturation process, that is intertwined with the stringency of sperm selection during natural conception. This provides new opportunities towards enhancing the safety and successes of ART, something of clear importance as the uptake of this service continues to spiral upward.

## Introduction

Infertility is a distressingly common condition affecting 15% of reproductive aged couples (Agarwal *et al.*, 2015). Since the birth of the first IVF baby in 1978, the number of people conceived by ART has grown at a rate few could have predicted, with over 9 million babies born globally by ART over the past 40 years (Kuhnt and Passet-Wittig, 2022). During this time, our understanding of human reproductive biology has advanced leading to important recognition that both partners contribute roughly equally to the burden of infertility. Indeed, it has been estimated that male factor infertility is the sole cause of ∼30% of all infertility cases and is estimated to be a contributor to 50% of overall cases (Agarwal *et al.*, 2015). While these data are perhaps not surprising, dismantling the myth that infertility is solely a female issue remains a persistent challenge (Kimmins *et al.*, 2024). The routine assessment of semen profiles has consistently revealed that humankind produces spermatozoa exhibiting extremely poor morphology and function, whereby infertile/sub-fertile men can be broadly characterized by the production of few ‘normal’ spermatozoa (Holt and Van Look, 2004). In fact, men presenting with fertility issues commonly exhibit low sperm concentrations among a sequelae of other diminished semen parameters including poor motility profiles and high levels of abnormal morphology (Kumar and Singh, 2015). Such defects contribute to an inability of sperm cells to traverse the female reproductive tract and have thus gained the status of traditional markers of poor sperm function. While such parameters do indeed correlate with male fertility, disappointingly, they have not proven to be definitive competency metrics. This situation presumably reflects the fact that, even after successful delivery to an egg, there remains a set of additional, tenacious barriers that sperm must surmount before successful fertilization can be achieved. It follows that we have much to gain from advancing our understanding of the fundamental sperm biology that underpins the process of natural conception.

From the perspective of a human sperm cell, the journey to natural conception begins with insemination into the anterior vagina, whereupon they compete with millions of counterparts to reach the ovulated egg. The intensity of this competition is reflected in the fact that only a small fraction of the inseminated sperm population (usually tens to hundreds) is able to successfully navigate the female reproductive tract to the site of fertilization (i.e. the distal end of the fallopian tube). This extensive attrition is attributed to an abundance of poor-quality sperm cells, a hallmark of even a normal or ‘fertile’ human ejaculate, and the sequence of highly specialized barriers these cells encounter within the female reproductive tract (Suarez and Pacey, 2006; Sakkas *et al.*, 2015). Layered on top of sperm functional characteristics, is the quality of the paternal genome and epigenome, which are arguably the most important components of each spermatozoon. Sperm populations harbouring diminished DNA quality have been studied for decades and have been implicated in numerous adverse reproductive outcomes including poor embryo quality, spontaneous abortion, and childhood diseases (Zini *et al.*, 2008; Avendaño *et al.*, 2010; Simon *et al.*, 2011; Lewis *et al.*, 2013; Alvarez Sedo *et al.*, 2017). Emerging evidence supports the notion that natural selection processes may have evolved to exclude such cells from participating in fertilization and passing on a potential corrupted paternal genome to the offspring (Pitnick *et al.*, 2020). Our advancing molecular understanding of sperm cell biology confirms that infertile men are also characterized by the prevalence of degraded DNA cargo in their spermatozoa (Zini *et al.*, 2001; Zeqiraj *et al.*, 2018). A critical reality, in the context of ART, is that a spermatozoon carrying a damaged genome can still fertilize an egg because it may not be entirely functionally compromised (Aitken *et al.*, 1998; Ahmadi and Ng, 1999).

Against a backdrop of falling male fertility (Mann *et al.*, 2020) and the disproportionate use of ‘high intervention’ ARTs used to treat this condition (Norman, 2022), is the potential risk of inadvertently using spermatozoa with compromised DNA integrity. Indeed, recent epidemiological data suggest a potential increased risk of birth defects and the perpetuation of defective semen profiles in children conceived via ICSI compared to that of their naturally conceived peers (Davies *et al.*, 2012, 2017; Belva *et al.*, 2016). While these data are not without debate, such observations raise the prospect that instigating conception with defective spermatozoa, including those with damaged genomes and/or altered epigenomes, may elevate adverse reproductive and general health outcomes for the next generation. Such risks highlight a pressing need to improve our current armoury of sperm selection tools to mitigate the unintentional use of poor-quality cells in assisted reproduction, with the ‘gold standard’ being the complex processes of natural conception itself. Thus, the sought-after improvements for artificial processes may only be achieved through advancing our knowledge of what makes a sperm cell capable of successfully navigating the female reproductive tract and fertilizing an egg *in vivo*. In this review, we consider the advantages and shortcomings of current, state-of-the-art sperm selection strategies and connect what we know about the sperm maturation process, which in concert, can offer new ways to improve ART outcomes by imposing selection criteria that reflect the stringency of the natural barriers sperm encounter prior to fertilization.

## Current sperm selection technologies

**Figure 1.**
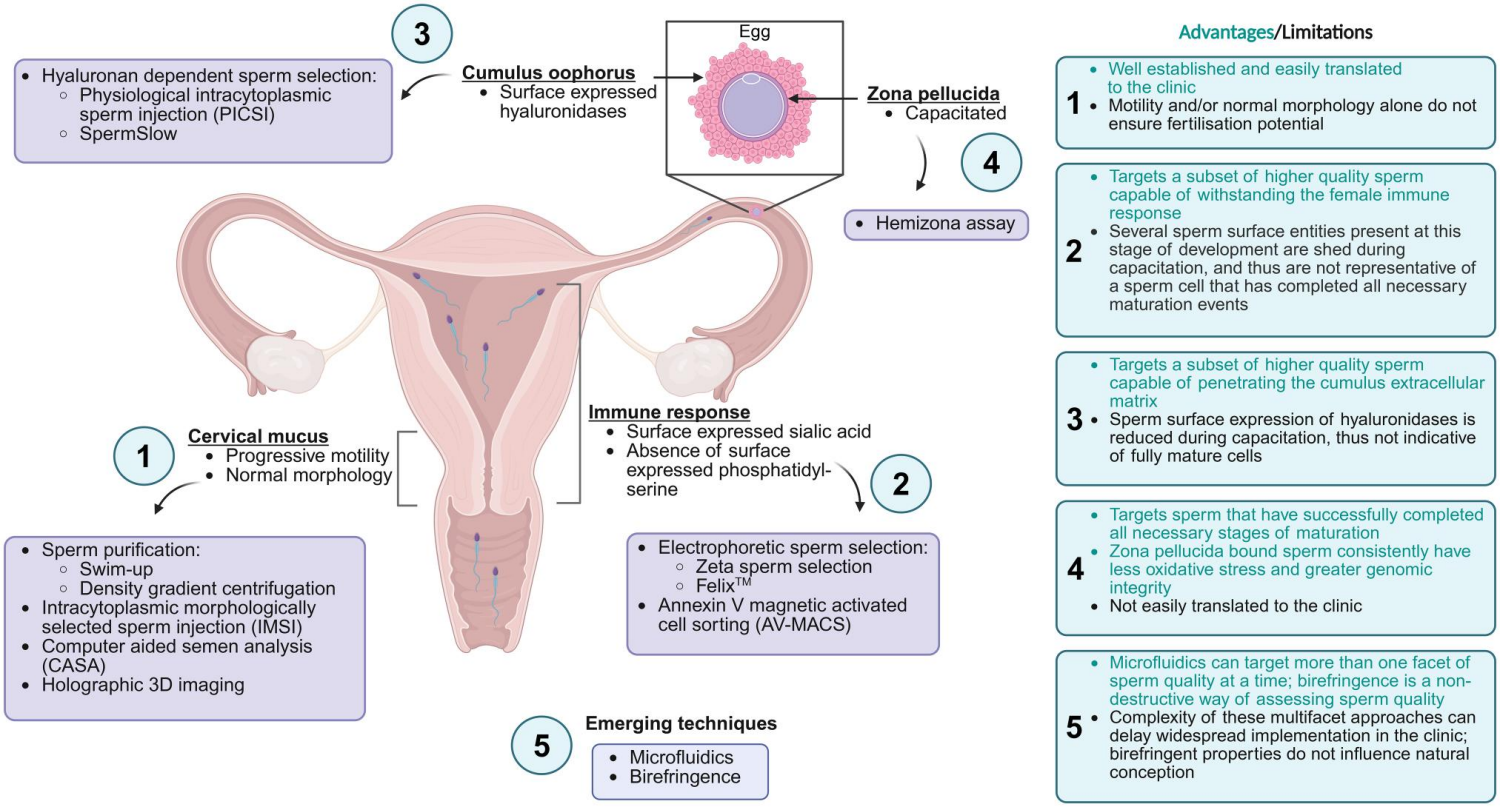
**The selection barriers spermatozoa encounter during natural conception, and advantages/limitations of the sperm selection techniques derived from these barriers.** As sperm transit the female reproductive tract, they encounter a series of natural selection barriers, each targeting different facets of sperm quality. Several of these aspects have been exploited for artificial sperm selection techniques (**1–4**). Advances in technology platforms have led to the development of methods that can target more than one facet of sperm quality, which more closely reflects natural sperm selection processes (**5**). Created in BioRender. De Iuliis, G.N. (2025) https://BioRender.com/f1y54jl.

Since the establishment of ART, numerous sperm selection techniques have been developed to isolate the sub-population of higher-quality spermatozoa from within the heterogeneous pool that characterizes a typical ejaculate. As expanded on below and summarized in (Table 1), such techniques have largely centred on the analysis of readily discernible characteristics of a spermatozoon including its motility profile, morphology, electrostatic potential, presentation of apoptotic markers, and/or ability to bind to physiologically relevant substrates such as hyaluronic acid (HA) and the zona pellucida (ZP). Figure 1 summarizes the selection barriers spermatozoa encounter within the female reproductive tract, and the advantages/limitations of the selection techniques derived from these barriers.

**Table 1.** Utility of current and emerging sperm selection techniques and analytical techniques, ordered from those with least potential benefit to the most, based on how well they reflect natural selection criteria, clinical outcomes, and risks involved.

Technique	Utility of technique	Limitations/reasons for caution	References	
Swim-up (SU) and density gradient centrifugation (DGC); *selection techniques*	Proven to enrich for motile spermatozoa with enhanced morphology	Sperm DNA damage is not always reflected in reduced motility and/or obvious morphological changes Exists a risk or propensity for selecting cells that contain fragmented DNA Mechanical shearing forces of centrifugation are linked to elevation in cellular reactive oxygen species production Metal content in colloidal silicon solutions can induce oxidative DNA damage	Aitken and Clarkson (1988) Henkel and Schill (2003) Agarwal *et al.* (2006) Aitken *et al.* (2014) Simopoulou *et al.* (2016) Muratori *et al.* (2019) Oseguera-López *et al.* (2019) Ogle *et al.* (2021)	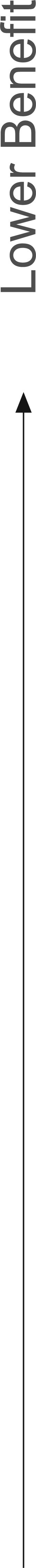
Annexin V-magnetic activated cell sorting (AV-MACS); *selection technique*	Removes sperm with apoptotic markers from an ejaculate Typically enriches for sperm with lower levels of DNA fragmentation When coupled with DGC, can significantly reduce miscarriage rate and increase live-birth rate	Exists a risk or propensity for selecting cells that contain fragmented DNA Contradicting evidence suggests externalized phosphatidylserine may play an important role in egg binding Lack of a clear consensus regarding impact on reproductive outcomes	Lee *et al.* (2010) Martínez *et al.* (2018) Oseguera-López *et al.* (2019) Pacheco *et al.* (2020) Ahmadi *et al.* (2022) Bibi *et al.* (2023)	
Computer-assisted semen analysis (CASA); *analytical technique*	Accurately measures sperm concentration, motility, and progressive motility Kinematics of swimming spermatozoa can predict fertility in the general male population	Sperm DNA damage is not always reflected in altered motion of swimming Minimal evidence to support or dispute clinical use	Larsen *et al.* (2000) Nixon *et al.* (2023) Tang *et al.* (2023)	
Holographic 3D imaging; *analytical technique*	Accurately differentiates between abnormal and normal sperm morphology and accurately measures sperm motility trajectories	Sperm DNA damage is not always reflected in altered sperm trajectories and/or obvious morphological changes Minimal evidence to support or dispute clinical use	Di Caprio *et al.* (2015) Dardikman-Yoffe *et al.* (2020) Kamieniczna *et al.* (2022) Wheeler *et al.* (2024)	
Motile sperm organelle morphology examination (MSOME); *analytical technique*	MSOME classification is correlated with DNA damage According to MSOME criteria: Incidence of morphologically normal sperm is positively correlated with ICSI fertilization rate Incidence of sperm with a morphologically normal nucleus is positively correlated with both fertilization rate and pregnancy outcome	Sperm DNA damage is not always reflected in subtle morphological changes Meta-analysis of 13 randomized controlled trials found very low-quality evidence to support clinical use	Bartoov *et al.* (2002) Pastuszek *et al.* (2017) Teixeira *et al.* (2020)	
Hyaluronan dependent sperm selection: Physiological intracytoplasmic sperm injection (PICSI); *selection technique* SpermSlow; *selection technique*	Effectively enriches for sperm cells with hyaluronic acid binding capability Proven to enrich for sperm cells with low levels of DNA fragmentation Selection based on Can increase fertilization rate, improve embryo quality and development, reduce miscarriage rate particularly in women aged over 35 years	A reduction in hyaluronidase enzymes on the sperm surface is indicative of a fully mature sperm cell Lack of high-quality evidence and consensus regarding impact on reproductive outcomes	Huszar *et al.* (2003) Nasr-Esfahani *et al.* (2008) Parmegiani *et al.* (2010) Lepine *et al.* (2019) Oseguera-López *et al.* (2019) West *et al.* (2022)	
Birefringence; *analytical technique*	Absence of birefringence is a proven indicator of DNA fragmentation Yields higher rates of fertilization, embryo development, and live birth rates in patients with complete asthenozoospermia	Minimal evidence to support or dispute clinical use Limited to cases of severe male infertility, i.e. studies fail to observe a significant improvement on reproductive outcomes in patients with progressively motile sperm Birefringence does not influence natural conception	Gianaroli *et al.* (2008) Ghosh *et al.* (2012) Garolla *et al.* (2014) Magli *et al.* (2023)	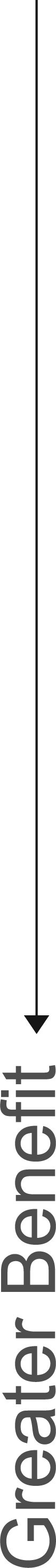
Electrophoretic sperm selection: Zeta sperm selection; *selection technique* Felix™; *selection technique*	Effectively enriches for sperm with greater negative charge Proven to enrich for sperm with low levels of DNA fragmentation When coupled with DGC, zeta sperm significantly increases the percentage of high-quality embryos and pregnancy rate	Low sperm recovery limits clinical use, especially for patients with oligozoospermia A reduction in sialic acid residues (i.e. a reduction in negative charge) on the sperm surface is indicative of a fully mature sperm cell	Ainsworth *et al.* (2005) Nasr Esfahani *et al.* (2016) Duarte *et al.* (2017) Karimi *et al.* (2020) Shapouri *et al.* (2023) Villeneuve *et al.* (2023) Hungerford *et al.* (2023)	
Microfluidic sorting; *selection technique*	Effectively mimics many of the female reproductive tract’s features Proven to enrich for sperm cells with low levels of DNA fragmentation Can increase the number of top-quality blastocysts and embryos	Minimal evidence to support or dispute routine clinical use Studies fail to observe a significant improvement in live birth rate	Yetkinel *et al.* (2019) Anbari *et al.* (2021) Keskin *et al.* (2022) Vasilescu *et al.* (2023)	
Zona pellucida binding; *selection technique*	Effectively enriches for morphologically normal sperm cells with low levels of DNA fragmentation Consistently yields higher quality embryos	Not easily translated to the clinic Minimal evidence to support or dispute clinical use	Liu and Baker (1992) Liu and Baker (2007) Paes Almeida Ferreira de Braga *et al.* (2009) Liu *et al.* (2011) Jin *et al.* (2016) Ganeva *et al.* (2021)	

### Selection of motile and morphologically normal spermatozoa

Immediately after insemination within the vagina, spermatozoa encounter cervical mucus, which begins to filter out cells with poor motility and concomitant abnormal morphology (Suarez and Pacey, 2006; Sakkas *et al.*, 2015). Given strong correlative evidence linking poor sperm motility and abnormal morphology with DNA fragmentation (Kvitko *et al.*, 2012; Sivanarayana *et al.*, 2012; Aydos *et al.*, 2015; Jakubik-Uljasz *et al.*, 2020), it is not surprising that some of the first sperm selection techniques developed for ART focused on this link, selecting motile and morphologically normal cells. Such techniques, including sperm swim-up (SU) and density gradient centrifugation (DGC), remain the stalwart for most andrological clinics today. For its part, SU harnesses the ability of motile spermatozoa to swim into the culture medium directly from the seminal plasma layered below or from a pre-washed pellet. By contrast, DGC partitions spermatozoa from semen based on their isopycnic density after layering the ejaculate onto a discontinuous density gradient (usually comprising a colloidal suspension of silica) and applying centrifugal force (Arcidiacono *et al.*, 1983; Simopoulou *et al.*, 2016). Under such conditions, the more dense, high-quality spermatozoa characterized by minimal retention of residual cytoplasm and possessing normal morphology, are isolated from that of lower-quality and immature sub-populations of less dense sperm cells. Not only have both methods been proven to enrich for motile spermatozoa with enhanced morphology, but they are also operatively simple and economic, making them an ideal choice for ART (Henkel and Schill, 2003; Simopoulou *et al.*, 2016; Oseguera-López *et al.*, 2019). However, there are concerns that when spermatozoa are placed under these artificial environments, there is an increased risk or propensity for selection of cells that contain fragmented DNA. Indeed, some individuals experience an increase in total sperm DNA fragmentation following DGC and SU (Muratori *et al.*, 2019). There are two likely explanations for this; the first being that sperm DNA damage, in general, is not always a characteristic reflected in reduced motility and/or obvious morphological changes (Avendaño *et al.*, 2009; Liu and Liu, 2013; Ogle *et al.*, 2021). Thus, morphologically mature, motile sperm cells harbouring DNA damage may not be effectively excluded during DGC and similarly, may be collected in the overlaying medium during SU. The second explanation is that the mechanical shearing forces of centrifugation inherent to both DGC and SU have been linked to elevation in cellular reactive oxygen species (ROS) production, which can lead to impaired sperm function and DNA damage across the entire sample (Aitken and Clarkson, 1988; Agarwal *et al.*, 2006). Additionally, the metal content in colloidal silicon solutions that are commonly used in DGC have also been shown to induce oxidative DNA damage (Aitken *et al.*, 2014).

In recognition of such limitations, there have been several attempts to improve the efficacy of sperm selection protocols based on the principles of motility and morphology. Such strategies include the adoption of computer-assisted semen analysis (CASA) systems to accurately measure the kinematics of swimming spermatozoa (Larsen *et al.*, 2000; Tang *et al.*, 2023), the detection of subtle morphological defects during the motile sperm organelle morphology examination (MSOME) (Bartoov *et al.*, 2002; Teixeira *et al.*, 2020), and more recently, the exploitation of high-speed off-axis holographic systems to map the three-dimension refractive-index profile of the sperm head, in tandem with the dynamic flagellum localization during free swim (Di Caprio *et al.*, 2015; Dardikman-Yoffe *et al.*, 2020; Gong *et al.*, 2021; Kamieniczna *et al.*, 2022; Wheeler *et al.*, 2024). While in theory, these techniques have the potential to improve reproductive outcomes, there is currently insufficient evidence to support a positive effect on clinical pregnancy and live birth rates, and it remains unclear whether these strategies are able to resolve the limitations associated with the continuum of motility profiles displayed by the fertilizing spermatozoon (Tung and Suarez, 2021; Zaferani *et al.*, 2021; Nixon *et al.*, 2023). An inherent limitation of these techniques is that the sperm motility profile is not static, and DNA damage is not always accompanied by detectable changes in sperm motility and morphology. Additionally, while the detailed, ‘strict’, assessment of sperm morphology is currently recommended by the World Health Organisation as a major component of the conventional semen profile (World Health Organization, 2021), there are major problems with this assessment criterion that have haunted its clinical application from the beginning. Apart from discovering methods to fix and stain cells in a way that adequately presents their important features with a minimum distortion, there is the fundamental issue of determining exactly what constitutes ‘normal’. It is clearly the case that human spermatozoa are not perfectly formed, but whether *in vivo* or *in vitro* is just not known. Furthermore, by confining the assessment of ‘normal’ to the very small number of perfectly shaped cells in the ejaculate, the advocates of strict morphology create statistical problems related to sample size and limited dynamic range. Given the intrinsic variability involved in the subjective assessment of sperm morphology, differentiating a fertile sample (with, for example, 6% normal cells) from an infertile sample (with, for example, 3% normal cells) would require the counting of many more than the recommended 200 spermatozoa to achieve adequate statistical power. So, while an overall assessment of sperm morphology may well reflect the quality of the underlying spermatogenic, or sperm differentiation and maturation process, the difficulties encountered in reliably quantifying this criterion from a diagnostic perspective, should always be borne in mind.

Overall, these data highlight a need to develop rational sperm selection techniques that more closely reflect the stringency of the female reproductive tract, i.e. molecular based techniques that go beyond defects that can be detected by standard microscopic evaluation by discriminating cells with low levels of DNA damage and functional maturity that mimics natural conception.

### Selection based on sperm membrane characteristics

It is becoming increasingly apparent that sperm membrane characteristics, including the expression of cell surface markers, can provide information regarding sperm maturation status and DNA quality. This comes as little surprise since spermatozoa must sequentially interact with cumulus cells, the ZP and the oolemma to achieve fertilization, with each of these interactions being governed by entities present on the sperm surface. In recent years, several sperm selection technologies have emerged that target sperm membrane characteristics by identifying surrogate molecular markers that correlate with DNA quality. One such marker, shared by many somatic cells and spermatozoa, is that of exteriorized phosphatidylserine residues, which represents one of the earliest signs that a cell is undergoing apoptosis (Schlegel and Williamson, 2001). There exists concerning evidence that while sperm apoptotic markers are negatively correlated with IVF fertilization rates, they are not always correlated with ICSI fertilization rates (Talarczyk-Desole *et al.*, 2016). Thus, during an ICSI cycle, an apoptotic sperm cell may be inadvertently injected into the egg with potentially adverse effects on embryo quality. Based on the principle that annexin V (a phospholipid-binding protein) is capable of binding phosphatidylserine with high affinity, strategies incorporating annexin V coated paramagnetic microbeads in conjunction with magnetic activated cell sorting (termed AV-MACS), have been devised to selectively remove apoptotic sperm cells from an ejaculate (Said *et al.*, 2006; Lee *et al.*, 2010). Spermatozoa that pass through the annexin V column (i.e. non-apoptotic sperm cells) typically have less DNA damage (Lee *et al.*, 2010; Ahmadi *et al.*, 2022; Bibi *et al.*, 2023). This promising technique requires further assessment to confirm if using the cells selected in this manner for ART can improve overall reproductive outcomes (Simopoulou *et al.*, 2016; Oseguera-López *et al.*, 2019). This is especially pertinent in view of evidence that, similar to DGC and SU, semen samples from some men have been reported to exhibit an increase in sperm DNA fragmentation after AV-MACS compared to the basal levels of DNA fragmentation recorded in the initial ejaculate (Martínez *et al.*, 2018). Potentially confounding this form of sperm selection, a recent study by Rival *et al.* (2019) not only reported the presence of phosphatidylserine on viable mouse spermatozoa but demonstrated that this class of lipids fulfil an important role in mediation of sperm–egg binding. While further studies are needed to determine if this phenomenon also holds true for human sperm–egg interactions, it nevertheless serves as a cautionary note that spermatozoa are highly unique cells and thus extrapolating knowledge based on somatic cell biology can be problematic.

As spermatozoa mature and traverse the male reproductive tract, they progressively become coated with a sialic rich glycocalyx that masks them from immune surveillance and is thus critical for their future survival as allogeneic cells in the female reproductive tract (Tecle and Gagneux, 2015; Ma *et al.*, 2016). Specifically, CD52, a highly sialylated glycosylphosphatidylinositol-anchored glycoprotein, is acquired during epididymal transit and thereafter forms a major component of the sperm glycocalyx (Kirchhoff and Schroter, 2001). Such sialic acid residues also impart a greater overall negative electrostatic charge to the mature sperm cell compared to that of immature cells (Giuliani *et al.*, 2004). Thus, adornment of sialic acid residues (and consequently, a greater negative charge) on the sperm surface has been identified as an important correlate of the completion of normal epididymal maturation. Exploiting this knowledge, two different approaches have been developed to select spermatozoa based on their electronegativity. The first isolates sperm cells that adhere to the surface of a positively charged test tube (termed, zeta sperm selection) (Chan *et al.*, 2006), while the second separates spermatozoa owing to migration towards a positive electrode within an electric field (i.e. electrophoretic sperm selection) (Ainsworth *et al.*, 2005). Importantly, sperm populations isolated using these techniques have been proven to harbour high DNA quality (Ainsworth *et al.*, 2005; Duarte *et al.*, 2017). In a clinical setting, certain modes of electrophoretic sperm selection have been shown to be as effective as (but not a significant improvement on) DGC in terms of fertilization rate, cleavage rate, and high-quality embryos while requiring a fraction of the processing time (6 min compared with 40–60 min) (Fleming *et al.*, 2008). Similarly, zeta sperm selection coupled with DGC offers a significant increase in high-quality embryos and pregnancy rate, compared to sperm selected by DGC alone (Nasr Esfahani *et al.*, 2016; Karimi *et al.*, 2020). Recently, the method of electrophoretic sperm selection was patented, and a commercial electrophoretic sperm isolation device (the Felix™ system) is currently being evaluated in several independent clinics. Emerging data demonstrate that the Felix™ device is capable of selecting sperm cells with less DNA damage than those selected by DGC, with the added benefit of being rapid and simple to use (Hungerford *et al.*, 2023; Shapouri *et al.*, 2023; Villeneuve *et al.*, 2023). However, the low numbers of sperm cells recovered following the application of both the Felix™ system and zeta sperm selection provides one drawback, which is particularly acute in the context of treating oligozoospermic males.

If a spermatozoon manages to evade the female immune response and the many other anatomical and physiological barriers they encounter during their ascent of the female reproductive tract, they next face the challenge of penetrating the cumulus–egg complex (Suarez and Pacey, 2006; Sakkas *et al.*, 2015). This complex serves as an important physiological selection barrier, with sperm capable of penetrating this barrier exhibiting better morphological characteristics and lower levels of DNA fragmentation than those that fail this challenge (Naknam *et al.*, 2019). One of the principal components of the cumulus extracellular matrix is HA (Zhuo and Kimata, 2001). It follows that the ability of mature spermatozoa to penetrate the cumulus layer is correlated with the surface expression of hyaluronidase enzymes, including sperm adhesion molecule 1 (SPAM1), which assist with cumulus dispersal (Lin *et al.*, 1994; Martin-Deleon, 2011; Seol *et al.*, 2022). In recent years, two dominant sperm selection techniques have been developed that take advantage of the HA binding properties of spermatozoa. The first of these features the use of HA immobilized to a solid support [termed, physiological intracytoplasmic sperm injection (PICSI)] and the other features HA suspended within a viscous medium. Despite these purification methods adopting physiologically relevant criteria and selecting sperm cells with low levels of DNA fragmentation (Huszar *et al.*, 2003; Nasr-Esfahani *et al.*, 2008; Parmegiani *et al.*, 2010), meta-analyses have concluded that current evidence is insufficient to support their ability to improve clinical pregnancy and live birth rates compared to conventional ICSI (Lepine *et al.*, 2019). However, re-evaluation of clinical trial data has raised the prospect that PICSI may be associated with reduced miscarriage rate, particularly in women aged over 35 years (West *et al.*, 2022).

Prior to fertilization, the surface architecture of a human spermatozoon undergoes a substantial capacitation-associated remodelling in preparation for egg interaction (discussed further below). This event involves the shedding of sialic acid to unmask proteins involved in fertilization (Ma *et al.*, 2012; Tecle and Gagneux, 2015), a reduction in surface exposed hyaluronidase enzymes, and a reciprocal increase in superficially exposed ZP receptors (Redgrove *et al.*, 2013). Thus, both electronegative- and HA-based systems may preferentially enrich for the subset of spermatozoa that have yet to complete capacitation. The preferential enrichment of non-capacitated spermatozoa may, in part, account for the lack of consensus regarding reproductive outcomes of sperm selected by electronegative- and HA-based systems, especially if the selection process is delayed well after semen processing, during which time high-quality cells may have already capacitated and thus potentially be discarded (Nixon *et al.*, 2023). However, as an important caveat to this interpretation, it has been demonstrated that electrophoretically isolated sperm cells retain the ability to capacitate normally under appropriate culture conditions (Ainsworth *et al.*, 2011).

The first contact between the male and female gametes is initiated upon adhesion of spermatozoa to the ZP; a resilient extracellular coat that surrounds mammalian eggs. It has become increasingly apparent that the ZP acts as a stringent sperm selection barrier, preferably binding motile spermatozoa with normal morphology, and importantly, with intact DNA (Liu and Baker, 1992, 2007; Ganeva *et al.*, 2021). While beyond the scope of this review, the ZP also provides a key species-specific barrier for gamete interactions, prohibiting spermatozoa from even closely related species binding to a heterologous egg (Hanada and Chang, 1972; Yanagimachi, 1972). Further emphasizing the importance of compatibility between the gametes, clinical studies suggest that only ∼14% of all motile spermatozoa produced by fertile men are capable of binding to the ZP, with this proportion dropping to only 4.3% in sub-fertile males (Liu *et al.*, 2003). These findings reinforce the limitations of sperm motility assessment as a relatively blunt determinant of sperm quality particularly in the context of selecting sperm cells for ICSI. It is therefore not surprising that clinical trials using spermatozoa selected for ICSI on the basis of adherence to human ZP have yielded significantly higher implantation rates and embryo quality compared to conventional ICSI protocols (Paes Almeida Ferreira de Braga *et al.*, 2009; Liu *et al.*, 2011; Jin *et al.*, 2016). Unfortunately, the mechanisms that govern the efficacy of sperm–ZP interactions remain to be fully elucidated and thus we have yet to fully realize the promise of harnessing ZP-based protocols for sperm selection to improve assisted reproductive outcomes (explored further below).

### Future directions/considerations for sperm selection

It is beyond doubt that sperm DNA quality and by extension, epigenetic and genetic integrity, is critical when it comes to determining the health trajectory of the offspring. It is therefore concerning that we lack the means with which to directly select a single sperm cell based on the quality of the paternal genome it carries. This situation contrasts with natural conception, during which the fertilizing spermatozoon appears to be selected in such a way that supports only those cells with the highest genomic integrity. To further optimize the safety and efficacy of ART, we therefore stand to benefit from the development of technologies that are underpinned by a deeper molecular knowledge of how sperm quality is stringently discriminated during their passage through the female reproductive tract.

A recent development in this field is microfluidics, a relatively young technology in its own right, combining various disciplines of science and engineering to provide a system that manipulates micro or nano litres of fluid in a precise manner. This technology platform has gained use in diverse fields, including medicine and physics, and its application for sperm selection is currently under evaluation. Microfluidic systems can mimic many of the female reproductive tract’s natural features and the corresponding response in sperm, including rheotaxis, thigmotaxis, thermotaxis, and chemotaxis (see the review by Ahmadkhani *et al.* (2022) for a more detailed overview on this technology). The use of such systems provides promise, with sperm selected in this manner generally exhibiting less DNA damage (Anbari *et al.*, 2021; Vasilescu *et al.*, 2023).

Another recent development towards selecting sperm with greater genomic integrity is birefringence evaluation; the splitting of a single ray of unpolarized light into two rays travelling in different directions. Birefringence is characteristic of cells with normal nuclear organization, and mature sperm heads exhibit birefringence because of the arrangement of nucleoprotein filaments into rods that are longitudinally oriented. Accordingly, the absence of birefringence is indicative of DNA fragmentation (Garolla *et al.*, 2014), and recent findings suggest sperm selection by birefringence may have a positive impact on ICSI outcomes, with the added benefit of being cheap, easy to reproduce, and non-damaging (Gianaroli *et al.*, 2008; Ghosh *et al.*, 2012; Ribeiro *et al.*, 2023).

We have already introduced the gatekeeper role of the ZP and its utility in differentiating the highest-quality spermatozoa, even among the already highly selected sperm population that can reach the site of fertilization during natural conception (discussed in more detail below). Without the technology to completely replicate the challenges encountered during a spermatozoon’s journey through the female reproductive tract, the biology of ZP–sperm interactions offers a promising solution to enhance ART outcomes. Indeed, clinicians such as Liu and Baker have recognized the potential of using a patient’s immature oocytes as a tool with which to harvest ZP-bound sperm for injection of their sibling mature oocytes (Black *et al.*, 2010). However, the finite nature and value of this resource is prohibitive to widespread adoption of such strategies in a clinical setting. As such, the advent of a suitable synthetic alternative, a ZP mimetic, could offer major advancements for the field. Towards this goal, two main areas of research need to be realized; the first being the complete characterization of the molecular basis of sperm–ZP binding (a summary of our current understanding is provided below), and the second being the advancement of biofabrication techniques to develop efficacious ZP mimetics. Fortuitously, one area of research rapidly progressing is the development of materials that mimic extracellular structures, holding potential for tissue engineering, regenerative medicine, and drug discovery (see reviews by Nicolas *et al.* (2020) and Aazmi *et al.* (2024) for more detail on the design and application of such mimetics). Synchronously, mounting interest has focused on characterizing the structural and chemical features of natural extracellular matrices, to the point that extracellular structural mimetics have been developed with a high degree of similarity, both in terms of structure and function, to those presented in the original tissue. We speculate that this technology could be used to develop ZP mimetics, alleviating the restrictions on using naturally occurring ZP and allowing for the widespread use in sperm-binding assays to isolate a population of high-quality spermatozoa prior to undertaking ICSI.

The production of such ZP mimetics is not feasible without first fully elucidating the molecular mechanisms governing sperm–ZP interactions. As discussed in more detail below, with the aid of emerging technologies such as mass spectrometry and gene editing, progress is being made towards resolving the molecular processes underpinning fertilization, including sperm maturation and sperm–ZP binding. In this context, recent headway has been made in the characterization of the sperm proteome at various maturational stages and comparing that of fertile and infertile men (Redgrove *et al.*, 2011; Ayaz *et al.*, 2018; Castillo *et al.*, 2019, 2023; Dias *et al.*, 2019; Panner Selvam *et al.*, 2019). Additionally, in conjunction with decades of research into the structural characteristics of the ZP, advanced gain-of-function and loss-of-function strategies have provided new understanding of the mechanisms by which sperm recognize and interact with the ZP (Baibakov *et al.*, 2012; Avella *et al.*, 2014). The continued characterization of key mediators of this interaction may unlock the means to develop novel sperm selection tools, which will ultimately help to deliver positive reproductive outcomes for patients undergoing ART in line with natural reproduction. Below, we discuss the current knowledge of the sperm–ZP interaction and how the biological principles underpinning this critical phase of fertilization may be harnessed to inform the development of novel technologies to discriminate functionally competent sperm cells harbouring high levels of genomic integrity.

## The molecular basis of sperm–ZP interaction

### Structure and function of the human ZP

The ZP is synthesized during follicular development in the primary follicle and surrounds the mammalian female gamete from this stage onwards including the oocyte (pre-ovulation), egg (post-ovulation), and preimplantation embryo until hatching of the blastocyst. The ZP plays several crucial roles in reproduction including supporting communication between oocytes and follicular cells (cumulus, granulosa, and theca cells) during oogenesis, ensuring species specificity of sperm adhesion, prevention of polyspermy, and providing protection to the egg and embryo prior to implantation (Wassarman *et al.*, 1999; Moros-Nicolas *et al.*, 2021). As a testament to the vital roles of the ZP, failure of normal ZP assembly, brought about as a consequence of mutation to genes encoding ZP proteins, results in female subfertility and infertility (Gupta, 2021; Li *et al.*, 2022; Wassarman and Litscher, 2022). First described in the mouse, the ZP was initially defined as being composed of only three glycoproteins, designated ZP1–ZP3. However, further characterization revealed that both ZP thickness and composition vary considerably between species, with the latter resulting from pseudogenization and duplication events of ZP genes (Moros-Nicolas *et al.*, 2021). Thus, it was discovered that the human ZP is composed of four glycoproteins, ZP1–ZP4, which are heterogeneously glycosylated with asparagine-linked (*N*-) and serine/threonine-linked (*O*-) oligosaccharides that are highly sialylated and sulphated (Dell *et al.*, 2003; Wassarman, 2008). When observed using high resolution scanning electron microscopy, the ZP appears as a delicate meshwork of thin interconnected filaments, but the precise arrangement of these filaments is yet to be fully elucidated (Familiari *et al.*, 1992, 2006). Using lectins and antibodies, and more recently mass spectrometric analysis, several ZP sugar residues have been reported including mannose, galactose, and sialyl-Lewis^x^ residues (Maymon *et al.*, 1994; Jimenez-Movilla *et al.*, 2004; Pang *et al.*, 2011; Gupta, 2021).

Both lectin-like and protein–protein interactions have been implicated as playing essential roles in human sperm–ZP binding (Clark, 2013; Tumova *et al.*, 2021). However, despite decades of research, the ZP glycoprotein(s), and the cognate ZP receptor(s) on the sperm cell surface that direct this interaction, remain elusive. Early studies performed in mouse by Wassarman and colleagues initially reported *O*-glycans attached to Ser^332^ and Ser^334^ of ZP3 as zona ligands for sperm binding (Florman and Wassarman, 1985; Chen *et al.*, 1998). This led to the generally accepted glycan release model, which proposes that following fertilization, cortical granules release a putative glycosidase that removes the *O*-glycans and thereby account for the inability of spermatozoa to bind to 2-cell embryos (Avella *et al.*, 2013). Both α-1,3 galactose and *N*-acetylglucosamine have been proposed as the sperm binding ZP ligand, however, mice lacking α-1,3 galactose or the putative sperm receptor for *N*-acetylglucosamine are fertile, albeit with reduced ZP binding (Thall *et al.*, 1995; Asano *et al.*, 1997; Lu and Shur, 1997). Furthermore, subsequent structural analysis of native mouse ZP proteins using mass spectrometry indicates that neither ZP3 Ser^332^ nor Ser^334^ are *O*-glycosylated (Boja *et al.*, 2003), and further, mutating the sites to prevent glycosylation has no observable effect on either sperm binding or fertility in transgenic mice (Liu *et al.*, 1995; Gahlay *et al.*, 2010; Avella *et al.*, 2013). Although the sialyl-Lewis^x^ antigen has been proposed to mediate human sperm–ZP binding (Pang *et al.*, 2011), more recent work has demonstrated that human sperm retain the ability to bind to transgenic mouse eggs expressing human ZP glycoproteins even after the loss of all sialyl-Lewis^x^ motifs (Avella *et al.*, 2014).

In pioneering studies, Dean and colleagues have taken advantage of gain-of-function and loss-of-function genetic engineering strategies to implicate ZP2 as the primary ligand responsible for the binding of human and mouse sperm to homologous ZP (Baibakov *et al.*, 2012; Avella *et al.*, 2014). Specifically, this team demonstrated the ability of spermatozoa to bind to the N-terminal domain of the ZP2 peptide, independent of ZP2 glycans. The model developed to account for these findings proposes that following fertilization, the ZP2 N-terminal domain is cleaved by ovastacin, an egg cortical granule astacin-like metalloendopeptidase encoded by *Astl*, thus preventing sperm from binding to 2-cell embryos. Mutation of the ZP2 cleavage site (^167^LA^↓^DE^170^) or genetic ablation of *Astl* maintains intact ZP2 and permit mouse spermatozoa to bind to the ZP even after fertilization and granule exocytosis (Gahlay *et al.*, 2010; Burkart *et al.*, 2012; Tokuhiro and Dean, 2018).

Despite these latest findings, the role of carbohydrates in sperm–ZP binding cannot be completely disregarded. Indeed, human spermatozoa pre-incubated with a variety of monosaccharides, complex carbohydrate moieties, and lectins, including D-mannose, D-galactose, and dextran sulphate, exhibit reduced binding to human ZP in hemizona binding assays (Oehninger *et al.*, 1991; Miranda *et al.*, 1997). Further, in a series of experiments by Clark and colleagues, a complex polysaccharide consisting of predominantly *O*-sulphated α-L-fucose known as fucoidan, was shown to inhibit sperm binding under hemizona assay conditions in a dose-dependent manner, without affecting sperm motility or their ability to complete capacitation (Mahony *et al.*, 1991; Oehninger *et al.*, 1992). It follows that a myriad of lectins and lectin-like proteins on the sperm surface have been demonstrated to have an affinity for the ZP and to be involved in sperm–ZP binding, including proacrosin, acrosin, zonadhesin, alpha-D-mannosidase, and arylsulfatase A (ARSA; see below) (Tumova *et al.*, 2021). While the irrefutable identification of ZP entities involved in sperm binding is critical for the future development of sperm selection tools based on the stringency of this interaction, the ZP is just one side of the equation, and equally important is the identification of entities on the sperm surface involved in ZP binding and understanding the mechanisms that give rise to fertilization competent sperm.

### Functional maturation of spermatozoa in preparation for ZP binding

**Figure 2.**
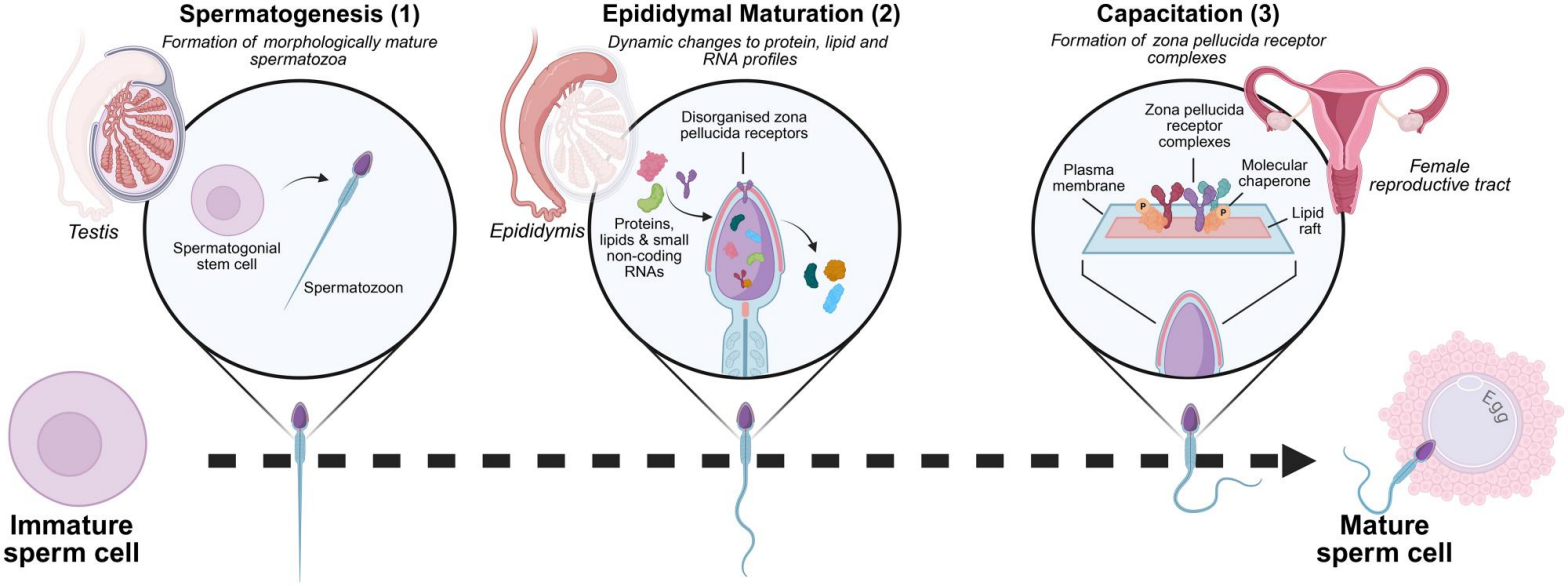
**Sperm maturation events necessary for zona pellucida binding.** In the testis, round spermatogonia differentiate into morphologically mature spermatozoa. Following spermatogenesis (**1**), functional maturity is progressively acquired during epididymal transit (**2**), where dynamic changes occur to the protein, lipid, and RNA profiles of the maturing spermatozoa. The final phase of sperm maturation, capacitation (**3**), occurs within the female reproductive tract. During this event, molecular chaperones are phosphorylated (P) and facilitate the assembly of zona pellucida receptor complexes on the sperm cell surface. Created in BioRender. De Iuliis, G.N. (2025) https://BioRender.com/n16vwfj.

Before mammalian spermatozoa can successfully interact with the ZP matrix, they must first undergo three distinct phases of maturation: spermatogenesis, epididymal maturation, and capacitation (Fig. 2). Despite being fully differentiated after the process of spermatogenesis (see Neto *et al.* (2016) for a detailed review), spermatozoa leave the testis as functionally incompetent cells, lacking progressive motility and the ability to bind to the ZP. Rather, much of the functional maturity is progressively acquired as spermatozoa transit the epididymis: a convoluted tubule that connects the testis to the vas deferens (Reid *et al.*, 2011; Sullivan and Mieusset, 2016). Anatomically, the epididymis is broadly subdivided into three major segments: the caput (head), corpus (body), and the cauda (tail) regions, with each having distinct roles in sperm maturation and storage. In most mammals, including humans, the potential to bind to the ZP is first demonstrated in spermatozoa isolated from the proximal corpus region (Saling, 1982; Moore *et al.*, 1983). Due primarily to their exceptionally compact genome, the balance of evidence suggests that spermatozoa are transcriptionally and translationally quiescent and thus, epididymal maturation appears to be extrinsically driven by the complex microenvironment the sperm cells encounter within the epididymal tubule (Zhou *et al.*, 2018). While the molecular basis of this maturation process is yet to be fully elucidated, emerging data suggest extracellular vesicles and molecular chaperone-laden ‘dense bodies’ within the epididymis deliver diverse cargo, including proteins implicated in sperm–ZP interaction, to the sperm surface (Bjorkgren and Sipila, 2019; Nixon *et al.*, 2019). More recently, global profiling of the proteomic changes associated with the epididymal maturation of mouse spermatozoa (Skerrett-Byrne *et al.*, 2022) documented that these cells shed over half their protein composition during their epididymal journey, suggesting that both the acquisition and loss of key proteins are necessary and potentially equally important for successful epididymal maturation. However, despite spermatozoa isolated from the cauda possessing the required machinery for ZP binding and fertilization, this machinery requires additional activation, which occurs during the capacitation process.

Capacitation represents the final phase of sperm maturation, during which sperm cells realize their full potential for fertilization (Aitken and Nixon, 2013). Capacitation is an intricate and tightly controlled process that occurs while spermatozoa ascend the female reproductive tract and includes characteristic changes in motility, acquisition of the potential for sperm–ZP interaction, and the acquired capacity to respond to such interactions by undergoing the acrosome reaction. In addition to these key physiological outcomes, capacitation is accompanied by an array of biochemical and cellular events including sterol oxidation and efflux from the plasma membrane, an increase in intracellular cAMP, upregulation of tyrosine phosphorylation, and a drastic remodelling of the sperm surface architecture in preparation for ZP adhesion (Reid *et al.*, 2011; Aitken and Nixon, 2013). Molecular chaperones, including heat shock 70 kDa protein 2 (HSPA2) and heat shock protein 90 (HSP90), feature prominently among proteins targeted for tyrosine phosphorylation during the capacitation of human spermatozoa (Ecroyd *et al.*, 2003; Redgrove *et al.*, 2013). Current models suggest that the phosphorylation of molecular chaperones initiates their active involvement in the formation of ZP recognition protein complexes and the positioning of these complexes on the sperm surface in preparation for fertilization (Asquith *et al.*, 2004; Bromfield and Nixon, 2013; Redgrove *et al.*, 2013). Although the molecular mechanisms underpinning this redistribution of sperm surface proteins are not well understood, an accumulation of evidence suggests that several cellular processes support this organization. For example, specialized membrane microdomains or lipid rafts have been demonstrated to contain a number of putative ZP receptors, which migrate during capacitation to the apical region of the sperm head: the cellular sub-region responsible for ZP interaction. Furthermore, these membrane microdomains exhibit specific affinity for the ZP (Tanphaichitr *et al.*, 2007; Boerke *et al.*, 2008; Nixon and Aitken, 2009).

It is important here to note that there is a strong argument for molecular redundancy in which sperm–ZP binding is achieved through the concerted action of a number of ZP receptor molecules, rather than via a simple lock and key mechanism akin to that involved in downstream sperm–oolemma adhesion (Bianchi *et al.*, 2014; Jean *et al.*, 2019). Indeed, the balance of evidence favours the idea that sperm–ZP interaction is mediated not by one or two molecules, but by the coordinated action of several sperm receptors that are brought to the cell surface and assembled into functional complexes during capacitation (Reid *et al.*, 2011; Tumova *et al.*, 2021). This may also explain why the pursuit of characterizing a single pair of partnering molecules has provided limited molecular insight towards our understanding of sperm–ZP interactions. Consistent with the multifaceted receptor concept, advancements in understanding the molecular mechanisms governing capacitation and subsequent ZP adhesion have led to the identification of a key sperm-enriched molecular chaperone, namely HSPA2, which forms the nucleus of several multimeric protein complexes residing in the peri-acrosomal region of the human sperm head (Redgrove *et al.*, 2012). One such complex has been shown to link HSPA2 with SPAM1 and ARSA. During capacitation, this complex appears to undergo a conformation change whereby SPAM1, likely having fulfilled its role in facilitating cumulus matrix dispersal via its hyaluronidase activity, is reoriented away from the sperm surface. Conversely, ARSA is translocated extracellularly and repositioned to the apical region of the sperm head where it is theorized to be involved in ZP adherence through its ability to act as a receptor for the sulphate groups which adorn the ZP glycoproteins (Redgrove *et al.*, 2013; Gomez-Torres *et al.*, 2021). HSPA2 appears to be intimately involved in orchestrating this dynamic remodelling, such that reduced expression of HSPA2 from the sperm proteome results in impaired sperm–ZP binding (Ergur *et al.*, 2002; Redgrove *et al.*, 2012).

### Factors linking defects in sperm–ZP interaction with sperm DNA damage

Within spermatozoa, normal physiological levels of ROS are essential for driving aspects of the capacitation cascade (Aitken *et al.*, 1998, 2015; Aitken and Nixon, 2013). However, if intracellular ROS production exceeds the antioxidant capacity of the cell, a state of oxidative stress (OS) ensues with negative consequences for cell quality. The term ‘antioxidant’ covers a broad range of factors, including small molecules and enzymes, which neutralize ROS and help protect against OS. While antioxidants are typically replete within the cytoplasm and mitochondria of somatic cells, spermatozoa have far less antioxidant capacity owing to the dramatic cytoplasmic depletion accompanying spermatogenesis and are thus rendered highly susceptible to OS. This situation is compounded by the presence of high levels of long chain polyunsaturated fatty acids within the sperm plasma membrane, which serve as effective substrates for lipid peroxidation (Aitken *et al.*, 1989; Walters *et al.*, 2018) and the perpetuation of peroxidation cascades and OS (Aitken *et al.*, 2012).

It has long been established that OS can lead to cellular lipid peroxidation and DNA damage. This in turn can lead to a loss of sperm motility and eventually cell death (Agarwal *et al.*, 2014). It is now also coming to light that other aspects of sperm function, not just DNA integrity, can be negatively influenced by OS. In a series of studies by Bromfield *et al.* (2015a), spermatozoa from infertile patients that failed to bind to the ZP were found to be deficient in HSPA2 [and BCL2-associated athanogene 6 (BAG6), a HSPA2-stabilizing protein] compared with fertile controls. Further, in spermatozoa from normozoospermic donors, ZP binding was significantly impaired following mild oxidative damage even though motility and cellular events associated with capacitation, such as tyrosine phosphorylation, were unaffected. Rather, the reduction of ZP binding was associated with impaired surface expression of ARSA, resulting from the peroxidation-mediated alkylation of HSPA2 with the lipid aldehyde 4-hydroxynonenal (4HNE) and consequent disruption of ZP receptor complexes (Bromfield *et al.*, 2015b). Bromfield and colleagues postulated that 4HNE protein adduction modifies the ATPase activity of HSPA2 and thus prevents it from fulfilling its role in protein trafficking or refolding events. Interestingly, these data also suggest that heat shock proteins are key targets for OS based 4HNE adduction, which in turn, can negatively impact the ability of affected cells to fertilize in the absence of any observable microscopic abnormalities. This aligns with clinical data indicating that as few as 14% of motile human sperm have the potential to bind to the ZP (Liu *et al.*, 2003). The vulnerability of molecular chaperones to 4HNE adduction has also been confirmed in numerous studies (Carbone *et al.*, 2004, 2005; Baker *et al.*, 2015). In addition to the direct impact OS has on mature spermatozoa, when this insult is encountered in the context of the testicular environment, it has been demonstrated to promote the ablation of HSPA2 in the developing germline, such that the resultant mature spermatozoa would also likely have diminished capacity for ZP binding (Bromfield *et al.*, 2017).

The formation and assembly of multimeric ZP receptor complexes offer a plausible explanation that, in part, accounts for the demanding maturation processes that sperm must undergo to reach functional competence. Sperm DNA integrity may persist only within the high-quality spermatozoa that have not suffered OS and can successfully complete the processes that lead to the assembly and presentation of receptor complexes necessary for productive ZP interaction, providing greater impetus for the use of ZP mimetics for the selection of high-quality sperm for ART. The link between DNA integrity and cell function aligns with the hypothesis that post-ejaculatory modifications arose through the selection of females to improve embryo quality through ‘sperm choice’. In other words, females dictate the required modifications of spermatozoa in order to distinguish high- from low-quality gametes (Pitnick *et al.*, 2020).

## Epigenetic considerations

While this review primarily focuses on the quality of the paternal genome, more recent data firmly implicate the importance of the paternal epigenome as a determinant of sperm, and subsequent embryo, quality (Lismer and Kimmins, 2023). It has long been established that epigenetic modifications including chromatin remodelling and DNA methylation play significant roles in spermatogenesis (Govin *et al.*, 2004; Gunes and Kulac, 2013; Hao *et al.*, 2019). What has more recently come to light is that in addition to the haploid genome, spermatozoa also deliver coding and non-coding RNAs to the mature egg (Ostermeier *et al.*, 2004; Jodar, 2019), which have been linked to blastocyst progression and embryo quality (Hua *et al.*, 2019; Trigg *et al.*, 2021; Hamilton *et al.*, 2024). The potential use of sperm mRNAs as predictors of ART outcomes is reviewed by Hernandez-Silva *et al.* (2022). Particularly relevant in the context of this review, it has been demonstrated that global DNA methylation levels are significantly lower in ZP bound spermatozoa than those manually selected by an embryologist for conventional ICSI (Wang *et al.*, 2021). The increased risk of methylation mutations during conventional ICSI has been putatively linked to a higher risk of autism in the resulting child (Wang *et al.*, 2021), further supporting a strategy that exploits positive ZP interactions for sperm selection during ART.

## Potential limitations of this review

While there is a strong rationale linking the stringency of natural conception processes to comparatively superior reproductive outcomes and subsequently healthier generations, this hypothesis is still under some debate, and so too is the significance of sperm selection directed by the quality of sperm maturation events and the ZP. We advocate for enhancing our fundamental cell biology knowledge of the complex processes that culminate in successful ZP–sperm interaction, however, clearly, other innovative strategies that rely on assessing sperm ‘fitness’ or morphologies for example, remain important pathways towards the same goal. It is remarkable that for such a defining process for organisms utilizing sexual reproduction, significant scientific unknowns remain for our and other species. Therefore, the exact definition of what reflects the best gamete quality remains elusive.

## Discussion

While fertilization and pregnancy rates are key metrics for the ART industry, the goal for all must be to produce subsequent healthy generations, while also providing family opportunities for people who struggle to conceive naturally. In the first half of this review, we provide an overview of the current state of sperm selection techniques for ART, calling attention to the need for new selection strategies aimed at ensuring genomic (genetic and epigenetic) quality of the cells selected in an effort to improve overall ART success rates, as well as long-term health outcomes for the children conceived. We specifically highlight the stringency of sperm selection enforced by the female reproductive tract during natural conception that cannot currently be replicated by our contemporary ART, which is reflected in inferior developmental success relative to natural reproduction (Sakkas *et al.*, 2015). Fittingly, the now incontrovertible evidence that these artificial processes are linked to elevated long-term health risks for the offspring, also reinforces this idea. Especially when you consider that they are particularly pronounced following ‘more invasive technologies’ such as ICSI. These data highlight a pressing need to improve our current armoury of sperm selection tools to improve the efficacy and safety of ART. We highlight that there is particular value in decreasing the use of ‘poorer quality’ cells for assisted reproduction. Specifically, we posit that this may only be achieved through improving our understanding of the processes that enable a sperm cell to successfully navigate the female reproductive tract and fertilize an egg *in vivo* so that we might harness this information *in vitro*.

Additionally, in the second half of this review, we provide a summary of our current knowledge of the molecular processes underpinning sperm maturation, with particular emphasis on capacitation, highlighting key knowledge gaps that may aid in the achievement of our aforementioned goal. In this vein, we strongly believe that upon the complete resolution of the entities governing sperm–ZP binding, i.e. the ligands adorning the ZP and the associated receptors on the sperm surface, ZP mimetics will become key contenders in the search for novel sperm selection strategies to significantly improve ART reproductive outcomes for all. This hypothesis is founded on the strong evidence linking the successful completion of capacitation to minimal OS and high DNA integrity within the sperm cell, and the promising studies utilizing the ZP to select high-quality spermatozoa. With the continued advancement of biofabrication techniques (particularly the development of extracellular matrices) and mass spectrometric analysis, devices that replicate the complexity of the sperm maturation process we have outlined, in concert with the intricate interactions and selection controls in the female reproductive tract and gametes, may be a reality for the not-so-distant future.

This review integrates current literature on artificial sperm selection with insights into the molecular processes underlying sperm maturation and sperm–ZP binding. In doing so, we highlight opportunities for advancement and underscore the ongoing relevance of addressing key knowledge gaps in sperm cell biology, particularly in shaping ART and improving offspring health outcomes: viewpoints that will resonate with both clinicians and reproductive biologists.

## Data Availability

No new data were generated or analysed in support of this review.
